# Supplementary material for: Prognostic Value of Carcinoembryonic Antigen (CEA) and Carbohydrate Antigen 19-9 (CA 19-9) in Gallbladder Cancer; 65 IU/mL of CA 19-9 Is the New Cut-Off Value for Prognosis
Source: Cancers (Basel). 2021 Mar 4;13(5):1089. doi: 10.3390/cancers13051089 (PMC7961941; doi:10.3390/cancers13051089)
Supplement: Supplementary file 1 [file cancers-13-01089-s001.pdf]

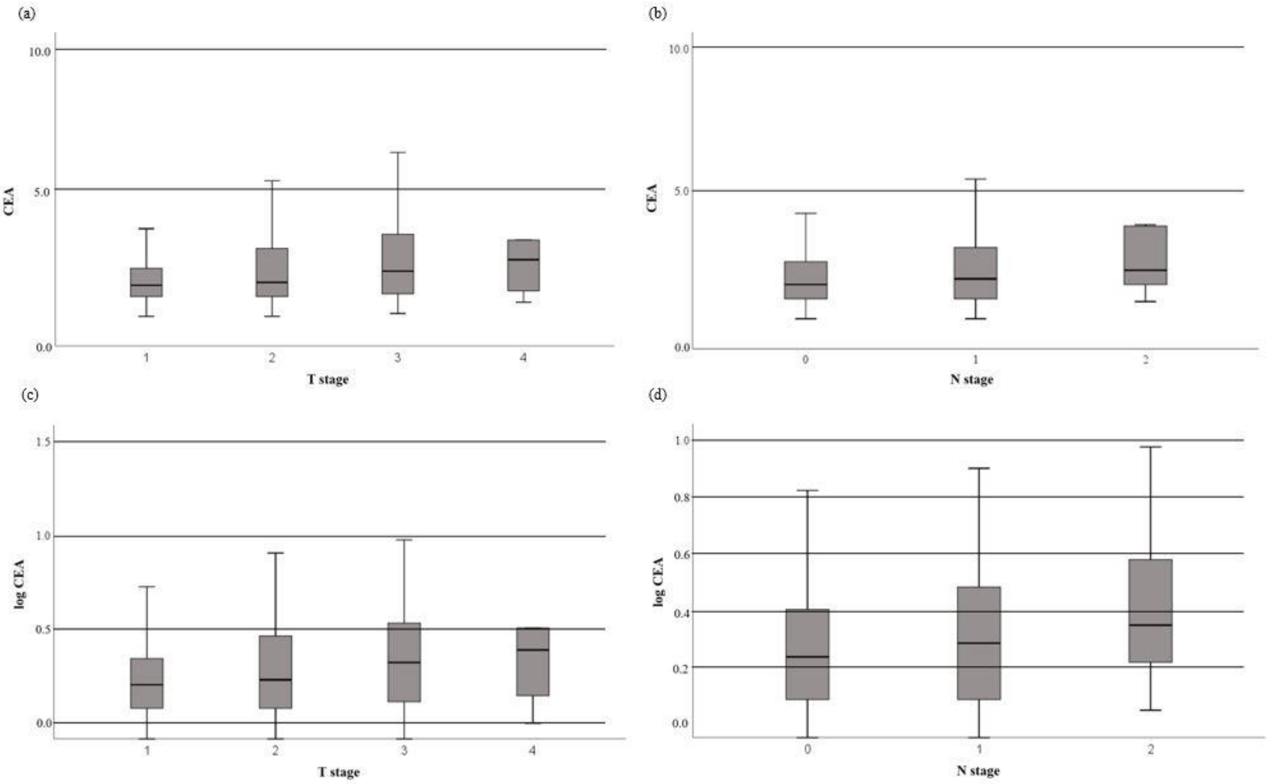

Figure S1. Distribution of preoperative CEA, logCEA level according to T and N stage. (**a**) Median CEA values at CEA were 1.80 IU/mL in T1 stage, 1.70 IU/mL in T2 stage, 2.10 IU/mL in T3 stage, and 2.50 IU/mL in T4 stage, respectively ($p = 0.016$). (**b**) Median CEA values were 1.70 IU/mL in N0 stage, 1.90 IU/mL in N1 stage, and 2.20 IU/mL in N2 stage ($p = 0.001$). (**c**) Median values of log CEA were 0.20 IU/mL in T1, 0.23 IU/mL in T2, 0.32 IU/mL in T3, and 0.39 IU/mL in T4 stages, respectively ($p = 0.001$). (**d**) Median values of log CEA were 0.23 IU/mL in N0 stage, 0.28 IU/mL in N1 stage, 0.34 IU/mL in N2 stage ($p = 0.015$).

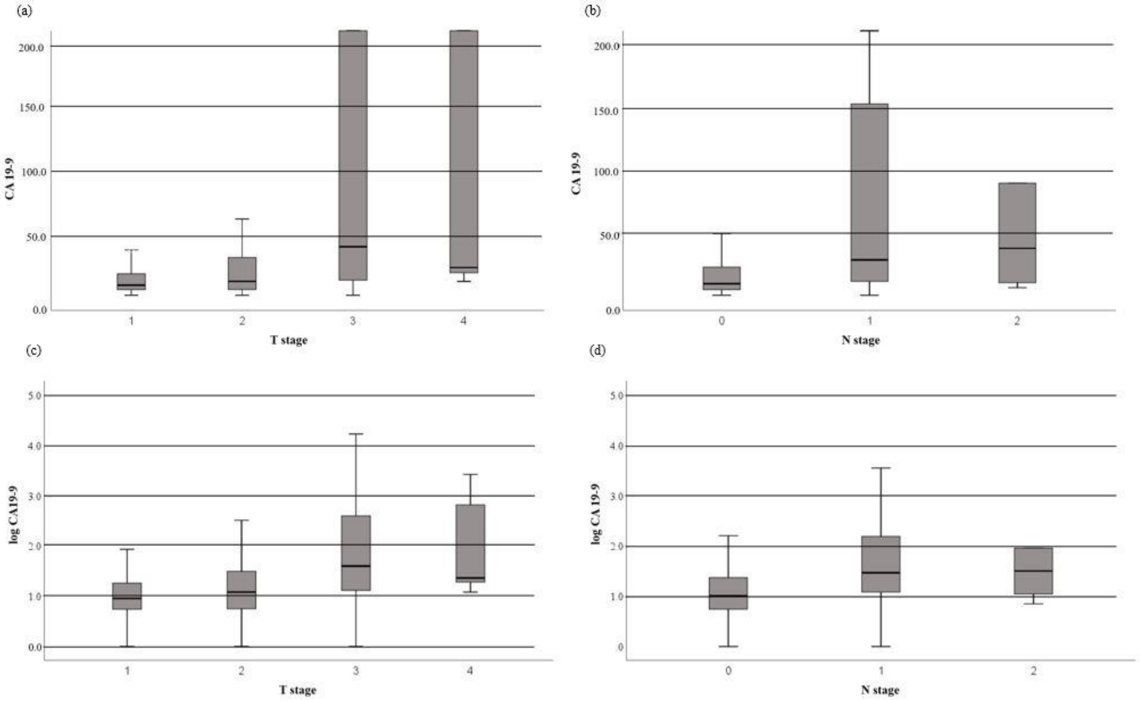

Figure S2. Distribution of preoperative CA19-9, logCA19-9 level according to T and N stage. (**a**) Median values of CA 19-9 were 9.00 IU/mL in T1 stage, 12.00 IU/mL in T2 stage, 39.55 IU/mL in T3 stage, and 23.00 IU/mL in T4 stage ($p = 0.894$). (**b**) Median values of CA 19-9 were 10.20 IU/mL in N0 stage, 29.10 IU/mL in N1 stage, 38.30 IU/mL in N2 stage ($p = 0.156$). (**c**) Median values of log CA 19-9 were 0.95 IU/mL in T1 stage, 1.08 IU/mL in T2 stage, 1.60 IU/mL in T3 stage, and 1.36 IU/mL in T4 stage ($p < 0.001$). (**d**) Median values of log CA 19-9 were 1.01 IU/mL in N0 stage, 1.46 IU/mL in N1 stage, 1.50 IU/mL in N2 stage ($p < 0.001$). The trend that elevation of both the levels of the tumor markers and T/N stage occurs simultaneously was inspected in the case of tumor markers, which was transformed as a log equation.
